# Molecular Application of Aptamers in the Diagnosis and Treatment of Cancer and Communicable Diseases

**DOI:** 10.3390/ph11040093

**Published:** 2018-09-28

**Authors:** Philisiwe Fortunate Molefe, Priscilla Masamba, Babatunji Emmanuel Oyinloye, Londiwe Simphiwe Mbatha, Mervin Meyer, Abidemi Paul Kappo

**Affiliations:** 1 Biotechnology and Structural Biochemistry (BSB) Group, Department of Biochemistry and Microbiology, Faculty of Science and Agriculture, University of Zululand, KwaDlangezwa 3886, South Africa; philisiwefmolefe@gmail.com (P.F.M.); presh4u@rocketmail.com (P.M.); tunji4reele@yahoo.com (B.E.O.); mbathalondy@gmail.com (L.S.M.); 2 Department of Biochemistry, College of Sciences, Afe Babalola University, PMB 5454, Ado-Ekiti 360001, Nigeria; 3 DST/Mintek Nanotechnology Innovation Centre, Biolabels Node, Department of Biotechnology, University of the Western Cape, Bellville 7535, South Africa; memeyer@uwc.ac.za

**Keywords:** aptamers, cancer, diagnostics, therapeutics, infectious diseases, SELEX

## Abstract

Cancer and infectious diseases such as Ebola, HIV, tuberculosis, Zika, hepatitis, measles and human schistosomiasis are serious global health hazards. The increasing annual morbidities and mortalities of these diseases have been blamed on drug resistance and the inefficacy of available diagnostic tools, particularly those which are immunologically-based. Antibody-based tools rely solely on antibody production for diagnosis and for this reason they are the major cause of diagnostic delays. Unfortunately, the control of these diseases depends on early detection and administration of effective treatment therefore any diagnostic delay is a huge challenge to curbing these diseases. Hence, there is a need for alternative diagnostic tools, discovery and development of novel therapeutic agents. Studies have demonstrated that aptamers could potentially offer one of the best solutions to these problems. Aptamers are short sequences of either DNA or RNA molecules, which are identified in vitro through a SELEX process. They are sensitive and bind specifically to target molecules. Their promising features suggest they may serve as better diagnostic agents and can be used as drug carriers for therapeutic purposes. In this article, we review the applications of aptamers in the theranostics of cancer and some infectious diseases.

## 1. Introduction

Infectious diseases such as Ebola, Zika, human immunodeficiency virus (HIV), tuberculosis, hepatitis, measles and human schistosomiasis as well as non-communicable diseases such as cancer are not only considered as life-threatening concerns worldwide, but they have also been declared as major causes of high mortalities and morbidities globally [1,2,3]. Although the human immune system, particularly the innate immune system, has devised means to combat host invasion and other antimicrobial actions through interferons and cytokines [4], the structural complexity and the mechanisms by which these etiologic agents replicate, assists them in impeding the actions of the innate immune defence system [5]. Hence, early diagnosis of these diseases enable prompt administration of treatment which in turn boosts immunity and prevent further viral progression, thus saving the lives of the infected. Meanwhile, existing challenges linked with these viruses include genetic evolution and resistance to treatment [6]; this requires extra channels to follow in alleviating their devastating effects. Available serologically-based diagnostic tools may not be that sensitive to detect these evolving viruses and parasites immediately after infection [7]. Nonetheless, studies have shown that aptamers are potentially good candidates for resolving issues pertaining to drug resistance, non-specific drugs and diagnostic tools with low sensitivity. Aptamers, which are short oligonucleotide sequences selected in vitro via the Systematic Evolution of Ligands by Exponential Enrichment (SELEX) process, are highly specific agents which bind to their targets with high affinity [8,9,10]. Their specificity and affinity-binding properties make them important for use in different applications [11,12]. Although aptamers possess similar binding affinities for their targets as antibodies, varying between nanomolar and picomolar (expressed as dissociation constants, i.e., Kd) [13,14,15], antibodies are still not only immunogenic but is also associated with high production costs. On the other hand aptamers show great supremacy due to their lack of immunogenicity [10,16,17] and synthesis which is done chemically at lower costs, their small sizes makes them versatile and enables them to pass through blood-brain barriers which cannot be achieved with antibodies [18,19]. Table 1, however, illustrates the strengths and limitations of aptamer technology. Due to the above mentioned advantages, aptamers have been applied in the diagnosis and treatment of various diseases such as HIV/AIDS (acquired immune deficiency syndrome), tuberculosis, hepatitis, Ebola, cancer, measles, Zika and schistosomiasis [20]. These diseases pose serious threats to human health because they have the ability to escalate and lead to fatalities if not diagnosed and treated early on. Therefore, the diverse characteristics of aptamers allows for their use in various applications ranging from disease diagnostics to bio-sensing and therapeutics [9,21,22]. Therefore, this review covers the importance of the molecular application of aptamers over other biological agents such as antibodies that are used in the diagnosis and treatment of the above-mentioned communicable and non-communicable diseases, and the role aptamers play in studies directed towards finding alternative solutions for the near future. 

## 2. The Synthesis of Aptamers

Aptamers have been widely explored and acknowledged for their structural versatility in the diagnosis and treatment of various diseases [23,24]. These short DNA or RNA sequences are selected through the SELEX process [24,25,26], and different SELEX methods have been described in literature and applied in several studies. Some of these include affinity chromatography and magnetic bead-based SELEX, nitrocellulose membrane filtration-based SELEX, as well as capillary electrophoresis-based SELEX [23,27,28]. The primary selection or conventional SELEX method of sequences often involves incubation of the selected oligonucleotide library with a specific target; these are usually proteins for therapeutic or diagnostic studies. Several rounds of amplification by polymerase chain reaction (PCR) then follow to screen for the most enriched pool, which can be identified by nucleotide sequencing, followed by functional aptamer synthesis [24,28,29,30]. The small sizes of aptamers, their ability to assume peculiar secondary structures and their strong affinity for their targets, qualify them for a variety of applications in various fields [31]. Their structural flexibility allows for post-SELEX modifications, which include substitution of the 2′-OH group of ribose with either a 2′-amino, 2′-fluoride or 2′-*O*-methylene [14], to enhance their stability properties and increase their half-life in biological fluids [12,13,32]. Such characteristics make aptamers more suitable agents for employment in theranostics over antibodies [16]. Since aptamer selection is carried out in vitro, their production is quick and cost effective, unlike antibodies, which require in vivo production [33] with time consuming and expensive down-streaming processes [34]. Aptamers have the advantage of being heat stable [35] meaning that they have the ability to denature and renature, while antibodies on the other hand, denature at high temperatures and lose their three-dimensional structures as well as their functionalities in the process due to the fact that they are proteins in nature [36]. Above all, unlike antibodies, aptamers are very sensitive and capable of distinguishing between isoforms of the same target hence they are regarded as good candidates for diagnostics and can be applied in therapeutics [37]. Furthermore, aptamer technology is continually advancing, that is SELEX processes are improving to ensure aptamer yields with minimal labour, making the process efficient and worthwhile [38]. 

## 3. Application of Aptamers in Cancer

Genetic mutations that occur during cell division predispose cells to cancer [42] and such may be caused by several external or even internal factors such as genes (hereditary), radiation, chemicals, smoking and old age. Under normal circumstances anticancer signalling pathways such as apoptosis eradicate cells with such mutations. However, the imbalances between cell growth and apoptosis can lead to carcinogenesis [43]. Once sensitized cancerous cells may divide and form aggregates in a particular part of the body, leading to the formation of malignant tumours, which if not detected or treated early, may further metastasize and become more difficult to control [44,45]. These cells may later migrate to other parts of the body, continue to multiply and successively impair the normal functioning of the affected area or organ [46]. Early diagnosis allows for early administration of therapeutic drugs thereby controlling the disease and giving affected individuals a better chance at survival [47].

### 3.1. Diagnosis

Genetic mutations in cancer cells can manifest in morphological, physiological and molecular changes, which may result in changes in the proteome. Differences in the cell surface proteome of healthy and cancer cells can be exploited targets (biomarkers) for diagnosis [48]. Conventional diagnostic methods and magnetic resonance imaging (MRI) involves tracking morphological changes of tumour cells to primarily diagnose cancers [49]. Conquering the ailment requires detection at a molecular level since conventional methods fail to detect cancer targets at this level [47,48]. Aptamers selected by the cell-SELEX process have shown great sensitivity in detecting cancerous cells as well as targeting cancer related proteins at an early stage regardless of their low expression levels, hence they are considered to be the best candidates for diagnosis at a molecular level [47]. Mechanisms by which DNA and RNA aptamers fold into outstanding nanostructures has piloted the development of a number of non-enzymatic isothermal circuits, to be used as molecular recognition tools, comprising of entropy-driven catalysis, hybridization chain reaction as well as catalytic hairpin assembly (CHA). RNA-based circuits have been noted for their sensitivity and this characteristic feature makes them potential agents for application in diagnosis and therapy, however, in vivo application of these circuits has been limited due to the lack of reporting (imaging) partners. Mudiyanselage and co-workers [50], devised a genetically-encoded catalytic hairpin assembly (CHARGE) method to design RNA-based CHA circuits, using split Broccoli as a reporter for sensitive RNA imaging in live cells. They focused on the CHA, which consists of two complementary nucleic acid hairpins designed in such a way that unprompted hybridization is kinetically delayed by inserting complementary regions within the hairpin stems [50]. The components of the CHA circuit comprised of two complementary RNA hairpins (H1 + H2), a target RNA (C) and a DFHBI-IT dye (a fluorescent dye). The H1 and H2 hairpins were modified with non-fluorescent Broc and Coli respectively, while the target C was used to catalyse and initiate H1 and H2 recombination which then activates DFHBI-IT thus enabling fluorescent signals to be recovered. To validate the fluorescing ability and sensitivity of these circuits, in vitro and in vivo studies were conducted, these demonstrated that CHA was able to detect RNA at very low concentrations with high sensitivity. These findings suggested a great potential of RNA-based circuits for use as molecular recognition tools and RNA imaging in live cells [50]. Additionally, the sensitivity of these circuits could be of paramount importance in the early diagnosis of cancer.

In 2010, Zhang et al. [48] reported a selection of aptamers specific to CCRF-CEM acute leukaemia cells. These aptamers were then conjugated to gold nanoparticles (AuNPs) and used in a colourimetric assay to detect cancer cells. Changes in the optical properties on the AuNPs in the presence of the biomarker resulted in detectable colour changes. Zhang and co-workers [48] further documented three other aptamers that were conjugated to quantum dots (QD), TTA1, AS1411 and MUC-1, producing different emission spectra in wavelengths ranging from 605, 655 and 705 nm respectively. When these aptamers were incubated with normal and diseased cells, QD-TTA1 showed selective visualization, which is highly dependent on the cell type. A clear spectrum was observed in C6 cells whilst in PC3, HeLa and NPA cell lines, a weak fluorescence spectrum was produced at the same 605 nm wavelength [48]. On the other hand, QD-AS1411 produced strong fluorescence on the cell membranes of all the cell lines at 655 nm wavelength. MUC-1 produced high fluorescence in HeLa cells at 705 nm and no fluorescence was produced by the QD-aptamers conjugated with normal cells that were used as negative control. Such results and behaviour showed aptamers have great selectivity and very high sensitivity, which are important factors to consider as far as early diagnosis is concerned [48].

Later studies by Wu et al. [51] reported the use of aptamers as sensors (aptasensors) that are constructed using fluorescence. Fluorescence based aptasensors are of great interest in disease diagnosis: they can be categorized into fluorescently labelled or label-free aptasensors. In many examples, the aptamer secondary structure switches induced by target recognition are suitably translated into a detectable fluorescent signal, producing an enhancement (“signal-on”) or a quenching (“signal-off”) effect, which directly reflect the extent of the binding with the target. For example, Shigdar and colleagues [52] chemically formulated a new small RNA fluorescent labelled aptamer (19 base DY-647) from a 40 base RNA aptamer isolated from a random oligonucleotide library, that has an affinity for epithelial cell adhesion molecule (EpCAM), which is upregulated in various cancer cells such as breast, gastric and colorectal cancer. The interaction of this RNA aptamer with EpCAM and subsequent internalization into the colorectal cancer cells was investigated using flow cytometry and confocal microscopy and the results showed successful binding and receptor-mediated endocytosis of this DY-647 labelled aptamer, suggesting that this aptamer can be potentially applied in the advancement of targeted therapeutics and molecular imaging agents for cancer diagnosis [52].

In a recent review Musumeci and co-workers presented an updated overview on fluorescent aptamers and aptamer-based fluorescence strategies discussing important cancer biomarkers such as PDGF, VEGF, angiogenin and mucin. They particularly focused on thrombin, which, even if marginally involved in cancer-related pathologies, is typically exploited as a proof-of-concept in sensing strategies’ validation. Indeed, thrombin is well known for its role in thrombosis as well as maintaining homeostasis of coagulation-related processes. Under normal health conditions, thrombin is found in very low concentrations in the blood. However, increased blood concentrations (pM to μM) of thrombin as a result of prothrombin proteolysis has been coexistent with diseases such as Alzheimer and cancer. Hence, thrombin is an important disease biomarker and a great target for diagnosis. The most studied aptamers of thrombin are the 15-mer and the 29-mer thrombin binding aptamers, able to non-competitively bind to different sites within the same protein, TBA_15_ binds to exosite I (the fibrinogen binding site) whereas TBA_29_ binds to exosite II which is the heparin binding site [32,41,53].

Wu and co-workers [51] conferred that aptamers amplified in line with Forster resonance energy transfer (FRET) and chemilluminescence resonance energy transfer (CRET) show high signal output and sensitivity which are properties that makes them important for applications intended to detect cancer biomarkers such as vascular endothelial growth factor (VEGF), platelet-derived growth factor (PDGF), tenascin-C, nuclear factor kappa-light-chain enhancer (NFκB) of activated B cells and prostate specific membrane antigen (PMSA). Hence, they are potential early cancer detectors and early diagnosis is imperative for the control and treatment of the disease [51].

### 3.2. Therapy

Among other cancer therapies, chemotherapy is administered as the primary treatment of cancer [54,55], however, treatment is dependent on the stage and the type of cancer and may alternatively be radiation, surgery or immunotherapy [56]. Excessive up-regulation of cancer genes such as the human epidermal growth receptor-2 (HER-2) causes disease (cancer) aggression that leads to certain chemotherapeutic resistance [26,57]. HER-2 is highly expressed in breast, ovarian and gastric cancers. Its ability to form heterodimers with other members of the epidermal growth receptor family, leads to HER-2 mediated signalling pathways that promote cell proliferation whilst inhibiting apoptosis, hence it is important in cancer cell survival [58] and for this reason it has been an important target for research towards cancer therapy [59]. Trastuzumab, an anti-HER-2 antibody has been used for the inhibition of HER-2 expression by binding to its juxta-membrane domain, leading to its down-regulation [60]. Trastuzumab also prevents heterodimerization between HER-2 and HER-3 resulting in restricted cell growth [61,62]. The use of trastuzumab in combination with chemotherapy has shown great efficacy in cancer treatment [63]. Despite the advantages that antibodies exhibit, their low accessibility and sensitivity along with high production costs and side effects (cardiotoxicity) have been reported [64].

RNA-based aptamers have recently emerged as promising gene and drug delivery agents for cancer therapy. This can be attributed to the many attractive properties that the aptamers possess, including, non-toxicity, large production capacity, and structural flexibility and amenability [10,17]. Early methods that have been used to design these delivery systems involved assembly of RNA with cargoes of interest, which unfortunately required the synthesis of long RNA molecules together with the cargo sequences. Recently, Wang and co-workers reported a new strategy known as ‘aptablocks’, which requires conjugation of the RNA complementary strand and the cargo, thereby forming an aptamer-stick and a cargo-stick. This method ensures appropriately-synthesized agents with reduced lengths, and sticky ends which enables reuse of the aptamers as universal delivery agents for various cargoes [65]. To validate the aptablocks method, the researchers developed an RNA aptamer tP19 conjugated to NGF siRNA. The tP19 is a pancreatic cancer-specific aptamer, while NGF siRNA is a known inhibitor of pancreatic cancer progression. In vitro testing of these conjugates in MCF-7 cell lines demonstrated the expected functionality of the aptamer-drug delivery agents [65].

The use of RNA-based aptamers as therapeutic agents or in targeted drug delivery systems, however, comes with its own hurdles, which includes the susceptibility to nuclease degradation. Fortunately, over the years several modifications have been implemented to overcome this. These include the incorporation of hydrophilic/hydrophobic polymers such as poly (ethylene glycol) (PEG) and poly (lactic acid) (PLA) with “stealth” or stabilisation properties to enhance the stability of aptamers to resist nuclease degradation as well as to delay renal excretion, thereby making RNA aptamers suitable for both in vitro and in vivo applications. Numerous in vitro and in vivo studies have been done to demonstrate the candidacy of aptamers and RNA aptamers in cancer therapy. For example, Gijs and co-workers [57] identified two aptamers (HEA2_1 and HEA2_3) that are specific to HER-2 by whole-cell SELEX using human breast cancer cells (SKBR3). Surface Plasmon Resonance Imaging (SPRi) showed that these aptamers have a fast association rate with a relatively slow dissociation rate. This makes them high affinity agents for HER-2 meaning that they can be used in the place of antibodies such as trastuzumab, performing the same function but with higher efficiency in drug delivery [57]. Reyes-Reyes and co-workers [66] demonstrated the effect of AS1411 aptamer (a multifunctional aptamer with G-quadruplex, which also serves as an anti-proliferative agent) [67] on cancer cells as it binds to nucleolin protein which is highly expressed and found mostly on the surface of cancer cells. Nucleolin is implicated in disease progression [68] and in cancer and it is known to limit Rac 1 (Ras-related C3 botulinum toxin substrate 1) signalling, thereby inhibiting cell death and promoting cell proliferation. However, they demonstrated that targeting nucleolin on cancer cell lines (DUI45, MDA-MB-468, A549 and LNCaP) with AS1411 limits nucleolin expression and promotes Rac 1 activation which in turn induces cell death through methuosis, hence preventing the proliferative activity of cancer cells [66]. AS1411 has been found to also function as a co-delivery system of doxorubicin (Dox) and shRNA to AGS adenocarcinoma cells. It is also noteworthy to mention that AS1411 showed great response in phase II clinical trials in renal cell carcinoma patients [14,32].

A study by Lin and colleagues has demonstrated that suitably modified aptamers are able to penetrate the blood brain barrier (BBB) and also overcome the reticuloendothelial system, hence, proving as optimal candidates in chemotherapy [69]. Other studies have shown that the use of aptamers in chemotherapy, in conjunction with therapeutic agents increases drug efficacy and selectivity to target only the diseased cells thus ameliorating drug side-effects [70,71,72]. For example, Pi and co-workers developed a DNA/RNA hybrid nanoparticle consisting of a phi29 pRNA three-way junction (3WJ) and an Endo-28 DNA thioaptamer embedded in its core [40]. The Endo-28 DNA aptamer target annexin A2, expressed in ovarian tumours, and is also found on the extracellular surface of endothelial cells. The pRNA-3WJ-scaffold is well-known for its exceedingly thermostable property. In a hybrid, it is known to facilitate proper folding of its binding nucleic acids without interfering with their target specificity. The endo-28-3WJ hybrid was tested for specificity against several cancer cell lines, with IGROV-1 and SKOV3 serving as annexin A2 positive cells and HEK293T cells used as negative control [40]. After incubation of Endo-28-3WJ with the cell lines and the elimination of non-specific binding by washes, flow cytometry was then used to determine whether Endo-28-3WJ nanoparticles were bound to the cells. The results showed high affinity of the nanoparticles to annexin A2 positive cell lines, with about 71.2% of IGROV-1, 51.7% of SKOV3 and only 17.3% of HEK293T cells binding endo-28-3WJ nanoparticles. These findings proved that pRNA does not interfere with the specificity of endo-28-3WJ to annexin A2. Additionally, pRNA-3WJ nanoparticles are non-immunogenic and their size enables them to pass through diverse biological barriers, hence they can penetrate cells, and this makes them ideal for in vivo targeted cancer therapy [40]. In this study, the authors further conjugated doxorubicin, an ovarian cancer drug, to the Endo-28-3WJ nanoparticle. Free Dox, Endo-28-3WJ sph1/Dox and Scr-3WJ-sph1/Dox (negative control), was tested against SKOV3 (annexin A2 positive) and HEK293T (annexin A2 negative control). SKOV3 cells treated with free Dox and Endo-28-3WJ sph1/Dox showed robust Dox fluorescent signal and the Dox conjugate is shown to be highly cytotoxic to SKOV3 cells and less effective on HEK293T cells after assessing cell viability using the MTT assay. These findings evidenced that Endo-28-3WJ sph1/Dox does not only target and penetrate cancer cells but can also deliver and release the cancer drug (doxorubicin) in specific cells without affecting healthy cells [40].

Binzel and co-workers [73] conducted an in vivo study using mice with prostate cancer to evaluate the antitumor activity of RNA nanoparticles constructed using the pRNA 3WJ core conjugated onto an RNA aptamer specific to prostrate membrane antigen (PMSA) overexpressed by prostate cancers. Results revealed successful and significant knockdown of miR17 and miR12 oncogenes, while increasing the expression of PTEN, a tumour suppressor. Ultimately, it was observed that tumour growth was repressed with very high efficacy at low doses [73]. Similarly, Pi and co-workers also conjugated three ligands with targeting abilities, (folate, a PSMA-binding 2′-fluoro (2′-F) modified RNA aptamer called A9g and the EGFR specific 2′F-RNA aptamer) to the 3WJ core and displayed these on extracellular vesicle (EV) surfaces in a bid to specifically target colorectal, prostate and breast cancer cells respectively. EVs are currently undergoing robust investigation as therapeutic carriers mainly due to their role in intercellular communication but however lack specificity. Results displayed successful delivery of siRNA and miRNA to cells and repressed tumour growth in three mouse models [74]. In 2014, Sanna and co-workers [75] reported on the formulation and development of the first polymeric aptamer targeted nanoparticles called BIND-014, which are currently in use clinically for chemotherapy. The nanoparticles were constructed using the SELEX technique and they comprised of docetaxel, a well-known chemotherapeutic drug as a payload, poly (ethylene glycol) PEG and PLGA, as amphiphilic co-polymeric core with stealth/stabilizing and buffering properties, and an RNA A10 aptamer as a targeting ligand, since it has a high binding affinity to PSMA overexpressed by prostate cancer cells [75]. The antitumor activity of these nanoparticles was demonstrated by Hrkach et al. [76] in rats bearing PSMA-expressing cancer cells and PSMA-negative cells where targeted (BIND-014), non-targeted and free docetaxel were used. The findings showed that BIND-014 was 10-folds more efficient in delivering docetaxel to PSMA-expressing cancer cells when compared to non-targeted nanoparticles and free drug. This indicated the significant role of aptamers in the formulation of specific drug delivery systems for prostate cancer therapy. Shu et al. [77] demonstrated the abilities of RNA-based nanotechnology in mice, using RNA nanoparticles with an 8-nt sequence corresponding to the seed region of the MicroRNA MiR-21, incorporated into pRNA-3WJ core and an epidermal growth factor receptor (EGFR) targeting aptamer which facilitates RNA nanoparticles’ internalization into cancer cells. MicroRNAs are responsible for regulating gene expression and cancer cell life cycle. Results demonstrated successful internalization of the highly stable RNA nanoparticles into cancer cells without accumulation in normal cells; the nanoparticles bound and inhibited MiR-21, which in turn reduced triple negative breast cancer (TNBC) progression [77]. These findings showed great potential of RNA-based nanotechnology application in improving cancer therapy.

Furthermore, the aptamers’ agonistic or antagonistic characters have made them to be superiorly applicable in cancer therapy [53,71]. Recently, Zhou and colleagues highlighted the screening of a single stranded FOXM1-specific DNA aptamer, to target and inhibit FOXM1 proteins. This inhibition was found to subjugate cancer cell proliferation [71]. FOXM1 controls a system of proliferation-related genes, its upregulation has been shown to initiate oncogenesis, as well as cause chemotherapeutic resistance in human breast cancer [78]. Platella and co-workers in their review summarized different G4-based aptamers as drugs with antiviral, anticancer, anticoagulant activity or with potential activity in other diseases (skeletal, prion and tuberculosis diseases). Among several anticancer G4-aptamers they also described the principal features of two anticancer G4-aptamers (T40214 and HJ24) [14]. T40214 is a G-rich oligodeoxynucleotide selected to target and inhibit the Signal transducer and activator of transcription 3 (STAT3) protein functions in different cancers [79]. STAT3 protein facilitates oncogenic signalling by mediating the overexpression of anti-apoptotic proteins Bcl-xL and Mcl-1, which in turn promote cell proliferation in cancers [14,79,80]. HJ24 is a DNA G-quadruplex aptamer which binds to Shp2 protein. Shp2 is a member of tyrosine phosphatases responsible for cell differentiation and oncogenesis, therefore is an important target for therapy. HJ24 binds and strongly inhibits Shp2 activity thus decreasing cancer progression.

## 4. Aptamer Application in the Diagnosis of Infectious Diseases

### 4.1. EBOLA

Ebola virus (EBOV) causes haemorrhagic fevers and is one of the most dangerous and deadly viral diseases [81]. The EBOV viral genome encodes seven viral proteins namely; nucleoprotein (NP), polymerase cofactor (VP35), VP40, glycoprotein GP, transcription activator (VP30), VP24 and RNA-dependent RNA polymerase L [82]. The negative stranded nature of this virus and the presence of small numbers of open reading frames in its sequence enable EBOV proteins to participate and act at different stages of the viral cycle thus promoting its propagation [81,83]. The Ebola virus’s VP35 protein enables the virus to escape host immunity by inhibiting the incitation of interferon regulatory factor 3 (IRF-3) production resulting from RIG-I (retinoic acid-inducible gene I) signalling. IRF-3 activation regulates the expression of interferons in response to viral infection [84,85]. Through its C-terminal domain (interferon inhibitory domain IID), VP35 binds to dsRNA and prevents its recognition by protein kinase R (PKR) [83,86], this in turn inhibits interferon production [87,88]. This precedes the rapid invasion of host cells and causes the accumulation of viral protein inclusions in the host cells’ cytoplasm [89]. This increases viral loads and results in devastating effects such as organ failure, disoriented fluid distribution as well as coagulation problems which lead to both internal and external haemorrhages in some cases [90].

EBOV threatens the lives of the infected and also places those taking care of the patients at risk [91]. EBOV is easily transmitted through aerosols or direct contact with any body fluids [92] either from an infected human or animal and hence precautions must be taken when handling infected patients as well as the corpses of those who have died as a result of the virus. Early diagnosis of Ebola remains a hurdle [93], not only due to its short incubation period inside the host, but also because of the widespread symptoms infected individuals exhibit, which are comparable and similar to symptoms caused by other viruses which are not so deadly. Sensitive diagnostic tools that make use of aptamers to detect the virus or viral proteins may be used to address this problem. In 2015, Wandtke and co-workers reported the identification of aptamers that contained conserved sequences rich in “GGGUGG” and “GAGGG” sequences in the loop region, a property that appear to be critical for the interaction between the aptamer and the antiviral protein. This information sequentially sets grounds for the design of molecular diagnostic tools capable of detecting Ebola, thus rendering aptamers better agents for use as diagnostic tools [94].

### 4.2. Human Immunodeficiency Virus

A competent immune system is critical to engage unwanted cell invaders such as HIV but this pathogen targets CD4^+^ T-cells, leukocytes, macrophages and dendritic cells [95], which perform key roles in immune defence. Viral invasion of these cells, subsequently leads to the uncontrolled replication of the virus which exhausts and cripples the immune system while simultaneously leaving the infected vulnerable to opportunistic diseases such as those caused by *Mycobacterium* spp.; this infection then later progresses to Acquired Immunodeficiency Syndrome (AIDS) [96]. However, early diagnosis is important even in asymptomatic individuals because it will bring about early drug administration which will impair the multiplication and assembly mechanisms of the virus, thus preventing the spread of the virus and delay its effects and allowing infected individuals to live an adequate standard of life, but also provide them a better chance at survival [97]. The viral proteins are critical targets for diagnostic and therapeutic purposes. Among other antigen detecting diagnostic tools such as the enzyme-linked immunosorbent assay (ELISA) and p24 antigen assays, studies by Tombelli et al. [9] demonstrated the successful binding of an aptamer-based sensor to Tat HIV protein, one of the gene expression regulatory elements that is essential to viral replication. The aptamer-based sensor was able to distinguish between Tat and Rev which proves that aptamers could be reliable diagnostic tools due to their design and the nature of their specificity [9].

### 4.3. Tuberculosis

*Mycobacterium tuberculosis* (*M.tb*) is the primary causative agent for tuberculosis (TB) and in 2015, approximately 10.4 million new cases and 1.8 million deaths were reported [98]. The infection begins with the penetration of the respiratory tract by the *Mycobacterium* spp. followed by the targeting of the alveolar macrophages, which are responsible for phagocytic immune defence mechanisms. Meanwhile, more immune defence agents including CD_4_^+^ T-lymphocytes and TγδS-lymphocytes producing specifically gamma interferons (IFN-γ), interleukins, tumour necrosis factor alpha (TNF-α) and macrophage colony-stimulating factor as well as other inflammatory inducing factors are produced to circumvent intracellular bacilli propagation. These defence agents stimulate macrophages and cytotoxic cells’ function to inhibit the growth of foreign organisms [99], thus restricting the bacilli within an infected cell and preventing its replication. However, evolution of *M.tb* has enabled the bacilli to overcome restraining immune effects by blocking reactive oxygen species (ROS) intermediates, triggering anti-inflammatory responses and further reducing phagocytic cell acidification [100]. Nonetheless, early diagnosis is the best at controlling the transmission of the bacterium as well as in allowing early administration of treatment. The skin reaction TB test has been used for a long time, and however, may turn out to be unreliable since it depends on the immune integrity in order to produce such reactions. On the other hand, other methods such as the sputum test, which are considered more reliable, have a long turn-around time. Diagnostic strategies aimed at detecting bacterial proteins such as MPT64, ESAT-6 and CFP-10 [101] can produce more reliable rapid diagnostic tests since these proteins are abundantly expressed during the early stages of infection, even in asymptomatic individuals. Recent studies conducted by Sypabekova et al. [102] demonstrated the identification of a MPT64 specific DNA aptamers. Surface plasmon resonance (SPR) demonstrated a strong binding affinity with a sensitivity and specificity of 91 and 90%, respectively. Such results provide evidence that aptamer-based detection methods could significantly improve the diagnosis of TB, provided they are approved for use in the market [102].

### 4.4. Zika Virus

Zika virus (ZIKV, ZIKAV), which is associated with birth defects such as microcephaly and other abnormalities in infants born to mothers infected during pregnancy, caused sporadic outbreaks in recent years [103]. Zika virus is an arthropod-borne virus that belongs to the *Flaviviridae* family and is primarily transmitted through mosquito bites by the *Aedes* spp. [104,105]. However, cases of mother-to-child, blood and sexual transmission have been reported [103,106]. ZIKV is related to viruses such as yellow fever (YFV), dengue (DENV) and West Nile (WNV) viruses [5,104]. Following its discovery in the Zika forest of Uganda where it was isolated from a sentinel rhesus monkey in 1947 [107], the virus has since then been reported in South East Asian countries while a recent outbreak that began in May 2015 until late 2016 was reported in the Americas’, particularly in Brazil. The escalation of ZIKV infections is associated with movement of people either due to urbanisation or tourism, which then contribute to the emergence and spread of the virus. Genetic changes in the viral genome are the basis for the difficulties leading to delays in diagnosis. Analytical studies carried out using a sequencing tool known as recombination development project (RDP), used in monitoring molecular evolution of the virus has shown that the virus has undergone at least 13 genomic recombinant episodes with observatory genetic breakpoints occurring only on the E (coding for the envelope) and NS5 which is the last non-structural protein of the seven genomic regions [108]. Viral evolution results in the emergence of several viral strains thus making vaccine development and viral detection using serological methods very difficult [109]. Due to the fact that *Flaviviridae* viruses other than ZIKA virus also produce similar symptoms, a distinct detection method is required for ZIKA, of which serologically-based tools do not qualify. Fortunately, studies have shown that aptamers are good for this purpose since they are able to detect and distinguish between similar viruses (dengue virus, West Nile virus and yellow fever). This proves them as the most reliable diagnostic agents, which could potentially offer an immediate response as soon as an outbreak begins, therefore allowing for the best control of infections [104].

### 4.5. Hepatitis Virus

Among other infectious diseases, hepatitis is responsible for the many of cases of liver inflammation, fibrosis and cirrhosis cases worldwide. The disease is divided into five types; namely A, B, C, D and E [110]. According to the World Health Organization (WHO), hepatitis A (HBA) and E (HEV) are transmitted through contaminated food and water as a result of poor sanitation or sanitary practices [111]. The detection of these viruses involves the use of microbial and plaque assays, serologically-based methods as well as PCR methods [112]. However, plaque assays exhibit low sensitivity since it requires high viral loads in a sample, which also imply that detection during the early stages on infection is difficult. Serologically-based methods on the other hand, rely on the manifestation of an immune response of which antibodies are not produced until the virus escalates. Although PCR methods are sensitive and definite they can only be performed in a laboratory setting and can therefore not be considered as rapid and low cost diagnostic methods. Type D (HDV) causes dual infection and occurs in patients infected with type B (HBV); a combination of the two may ultimately lead to life threatening consequences if not treated. Type B and C (HCV) are the most prevalent, with an approximately 96% mortality rate [113]. According to the Center for Disease Control (CDC), type A and B are transmitted through contact with infected blood, which includes exposure due to receiving infected transfused blood, as well as through unprotected sexual activity. Mother-to-child transmission has also been reported in type B [114] and has been noted for its distinct mechanisms of replication which involves the engagement of protein-primed transcription of an RNA intermediate, the pre-genomic RNA (pgRNA) occurring within viral capsids. The virus’s pgRNA packaging, together with reverse transcriptase (RT) (also known as the P protein), is important for the assembly of viral capsids [115].

Worldwide, chronic hepatitis is mainly caused by hepatitis C. Its positively-stranded RNA encodes for one polyprotein which requires post-translational processing as well as the involvement of proteases to cleave it into its functional components which comprise of both structural and non-structural proteins. The core protein C and the two envelope proteins (E1 and E2) comprise the structural proteins whereas NS2 (Protease), NS3 (Serine protease, helicase), NS4A (Serine protease cofactor), NS4B and NS5A proteins and NS5B, an RNA-dependent polymerase and an enzyme that is critically important for HCV replication [116], make up the non-structural proteins. The mechanisms by which the viruses (HBV and HCV) replicate themselves and the elements of these mechanisms are important targets for diagnostics. There is an increasing need for rapid diagnostic tools that are specific enough to detect infection by tracking the viral components even in asymptomatic patients [117]. Aptamers are the best candidates for this since they present high specificity and affinity for their targets. Mirian and co-workers [117] reported the advantages of using aptamers as tools for the development of biosensors capable of detecting the hepatitis B virus and described the identification of an RNA aptamers (HBs-A22), which was used for the detection of HB surface antigen (HBsAg) that is expressed on the surface of infected hepatoma cells (HepG2.2.15). Furthermore, Mirian et al. [117] documented that fluorescein isothiocyanate (FITC) tagged HBs-A22 and fluorescence microscopy was used to demonstrate binding to HBsAg expressing cells. A cyanine3 tagged RNA aptamer was used to detect HCV core-protein using a chip-based assay. An aptamer selected against HCV E2 glycoprotein was used in an enzyme linked apto-sorbent assay (ELASA) was used in qualitative and quantitative analysis of viral particles. These studies showed the extensive and potential use of aptamers as tools for the early detection of viruses even during the window period [117].

### 4.6. Rubella Virus

Rubella virus (RV) is etiologically responsible for causing German measles. This virus consists of a positive sense RNA genome, three structural proteins and two non-structural proteins namely the capsid and the glycoproteins E1 and E2; with P60 and P150 respectively, all of which are involved in the virus life cycle and replication and are important targets for vaccine and drug development [118]. Rubella is a highly transmissible virus, the only one from the genus *Rubivirus* of the *Togaviridae* family [22]. Its mode of transmission is mainly by inhalation of air droplets produced when an infected person coughs or sneezes [119]. Mother-to-child transmission has also been reported and may lead to deleterious effects such as miscarriages, foetal defects known as congenital rubella syndrome (CRS) [120,121], which may cause heart defects, thyroid dysfunction, loss of hearing and other conditions including autism. Rubella symptoms appear from at least 2 to 3 weeks after infection and these include; mild fever, red rashes, joint pains, headaches and enlarged neck lymph nodes. Moreover, the symptoms alone cannot be used for definite diagnosis; hence, confirmation by laboratory tests, which detect antibodies (i.e., serological assays), or the detection of viral RNA (using PCR) is required. In 1997, Revello and colleagues employed reverse transcription (RT)-PCR in the detection of prenatal RV. Among other specimens tested, it was concluded that the amniotic fluid (AF) was more suitable for the detection of prenatal RV. Regardless of the sensitivity of RT-PCR, it was also found that false negative results might be obtained when the test is done immediately after maternal viral infection [121]. Hence, Mori et al. [122] develop a novel reverse transcription loop-mediated amplification (RT-LAMP) method for the detection of RV. Once tested using RT-PCR on isolates sensitive to the Takashi vaccine strain, RT-LAMP was found to be as sensitive as RT-PCR since it detected infection with more or less the same specificity as the well-known RT-PCR. Nevertheless, PCR based methods are performed only in the laboratory by experts and for this reason it is not a readily available method and cannot always be employed in clinical settings. Moreover, despite the use of serologic assays, viral detection may be delayed because antibodies are not produced until after a certain period, also known as the window period or seroconversion. Therefore, the need for sensitive, specific and easily applicable tools such as those that are aptamer-based is currently mounting.

### 4.7. Human Schistosomiasis

Schistosomiasis (commonly known as bilharzia) is a disease that is caused by parasitic blood flukes or trematodes called schistosomes. *Schistosoma* species, namely *Schistosoma haematobium*, *S. mansoni* and *S. japonicum* are the main causes of human schistosomiasis [123]. Disease transmission to the human host is by means of direct contact with cercariae that are released by snails found in open water sources. Once within the human host, cercariae changes into schistosomulae which resists immune responses and enters the blood stream where the parasite lays its eggs and then migrate to different parts of the body, mainly in the urogenital area, liver, lungs, heart, kidneys and intestines [124]. Eggs hatched from adult worms are then excreted through faeces and urine and enter open water sources, were they then hatch into miracidia and quickly penetrate host snails to become sporocysts that develop into cercariae via asexual reproduction [125]. Early diagnosis of the disease is important for the control of disease escalation which may lead to deleterious effects that predispose infected persons to cancer and lead to the damage of major body organs [126]. However, low intensity of the infection may escape detection by diagnostic tests such as the Kato-Katz smear methods resulting in false negative results [123,127]. Serologically-based methods can also be used for the detection of Schistosoma. However, these diagnostics tests cannot differentiate between infections with trematodes different *Schistosoma* species or other helminths such as cestodes and nematodes, and therefore have low specificity for the detection of helminth infection. This highlights the need for extremely sensitive and specific agents such as aptamers for the detection of schistosomes [123]. Long and co-workers studied two DNA aptamers; namely LC6 and LC15. These were tested against *S. japonicum*, *S. kipo*, *Fasciopsis buskis* and *Enterobus* eggs and it was shown that both aptamers have strong affinity only for *S. japonicum* eggs. The tissue imaging results clearly showed that LC15 could strongly bind to *S. japonicum* eggs in the liver [123].

## 5. Applications of Aptamers for Viral Therapy

Despite the fact that there has been a huge interest in aptamers and that countless scientific studies have been published on the topic, the full potential of aptamers in diagnostics and therapeutics have not yet come to fruition. The approval of Macugen^®^ (pegatnib sodium) an anti-vascular endothelial growth factor (VEGF) aptamer by the Food and Drug Administration in 2004 [15] for the treatment of age-related macular degeneration is a great milestone and demonstrates the potential of aptamers [14,37,53,128,129]. Since then, many more useful studies have been piloted in the search for solutions for the treatment of HIV, TB, ZIKA, Ebola, cancer, hepatitis and measles. The use of nanostructures in targeting biomolecules for diagnosis and therapy is increasing; in vivo RNA nanotechnology leads the way in this regard [76,130,131]. This is due to the fact that RNA and proteins are enzymatically synthesized by transcription and translation of analogous gene sequences and the occurrence of RNA nanostructures seen to be interacting with proteins has been found in nature involving ribosomes, spliceosome, RNase P [130]. 

Schwarz-Schilling et al. [130], has shown that the interaction of RNA with proteins can be mediated by aptamer motifs which have been used in identifying RNA molecules with fluorescent proteins or enzymes. Developing RNA-protein nanostructures involves co-expression of RNA and functional constituents (aptamer motifs, proteins or enzymes) in one reaction [130]. However, this co-expression of multiple aptamers specific for various biomolecules can be a major challenge, since the binding affinities and expression levels of the proteins may vary. Schwarz-Schilling et al. [130], used a 40 nt pRNA 3WJ as the core motif of an RNA-protein nanostructure, which incorporated four aptamers (TAR, PP7, streptavidin and malachite green); two of the aptamers bind specifically to fluorescent proteins (Tat and PCP), one to streptavidin and the other to malachite green (MG). With the aid of fluorescence from the MG aptamer and Förster resonance energy transfer (FRET) between the fluorescing protein molecules, the authors were able to study the binding parameters and the stability of the complex nanostructure. The TAR is a 28 nt aptamer selected to bind tat (a transcriptional activator) from bovine immunodeficiency virus (BIV), the PP7 is a 25 nt long aptamer that binds to the amino acid sequence of the peptide phage PP7 coat protein. Added to this, Schwarz-Schilling and co-workers investigated RNA stability in a cell-free expression system using crude cell extract (previously used for the assembly of protein filaments or virus particles) from *E. coli*. After studying the aptamers at different positions, it was concluded that the RNA was stable and suitable for use as a scaffold. The co-expression and localization of the RNA-protein complex was investigated in vitro in a one-pot reaction using streptavidin beads and the expression of the RNA-protein structure was investigated inside the bacteria. This study clearly indicated that in vitro ‘cell-free’ expression is preferably the best route, since it does not require extra measures required for optimizing in vivo RNA-protein expression and provides better expression under controlled conditions. These results are evidence to the advancing RNA-based technology, which not only ensure high efficacy of expression, but a better yield under controlled conditions in vitro. This is a great improvement, which is important in the use of RNA aptamer for both diagnostics and therapy [130].

### 5.1. Ebola and Zika

In vitro studies by Binning and co-workers [83] identified two aptamers (1G8-14 and 2F11-14), which bind specifically to the interferon inhibitory domain (VP35IID) of VP35 EBOLA protein. Such findings certify that these aptamers may be applied as inhibitors of the dsRNA binding to VP35 and therefore therapeutically prevent the spread of the virus. Subsequently, Wandtke et al. [94] reported that aptamers with specific binding ability to the zinc-finger functionally prevent Ebola virus replication.

### 5.2. Aptamers in HIV Therapy

Despite the availability of efficient treatments for both HIV and TB, the development of drug resistance is growing problem and hence, alternative treatment for HIV and TB is needed [132]. Presently, the FDA has approved anti-retroviral regimen which includes nucleoside reverse transcriptase inhibitors (NRTIs), non-nucleoside reverse transcriptase inhibitors (NNRTIs) and protease inhibitors (PIs). Although these are not curative agents, they do however suppress viral replication [95]. Drug resistance and the presentation of severe side effects is an apprehensive issue that signifies the need for other means of drug delivery by suitable agents such as aptamers, which are highly specific and have been considered for use as co-operative agents of chemical anti-retroviral drugs [97]. Several studies have demonstrated the effectiveness of nucleic acid-based aptamer synthesis against important HIV components such as gp120, reverse transcriptase (RT), integrase (IN) as well as long terminal repeats (LTRs) and untranslated regions (UTRs).

#### 5.2.1. Aptamers Targeting gp120

The HIV-glycoprotein 120 (gp120) is found on the surface of the HIV virus and initiates host invasion as it binds on CD4^+^ cell receptors, which is a critical step in HIV infection. Viral entry inhibitors like Maraviroc T-20, which have been approved by the FDA and are currently in use, can counter HIV entry. However, due to the development of drug resistance other options are being investigated. Zhou et al. [133] synthesized anti-gp120-siRNA chimera (A-1 and B-68) and tested the aptamers’ affinity in comparison to a B4ot77 aptamer discovered by Dey and colleagues [134] on HIV-1BaL gp120 protein. The results demonstrated that both aptamers could tightly bind and inhibit gp120 at different regions and epitopes of the protein, hence preventing viral entry [134,135]. Additionally, this study revealed that aptamers could be used as carriers for dicer substrate siRNA, which inhibits viral replication and infectivity in cultured CEM T cells and primary blood mononuclear cells.

G-quadruplex-forming oligonucleotides have in recent years come onto the scene as potent and novel aptamers. Their three dimensional assembly and non-canonical DNA structure confers on them the ability to recognize and target proteins involved in gene expression during the life cycle of the HIV virus, which include gp120 [136]. Present on gp120 is a co-receptor binding site known as the V3 loop, which is imperative for viral entry. The phosphorothioate 8-mer d(5′TTGGGGTT3′) commonly known as ISIS5320 was the first G-quadruplex antiviral agent with the ability to bind the V3 region of the gp120 envelope by forming a rigid tetrameric parallel-stranded structure that inhibited viral entry and cell fusion at sub-micromolar concentrations [95,137]. Gonzalez et al. [95] reported the discovery and modifications of a 6-mer d(5′TGGGAG3′) known as the Hotoda sequence, which also has anti-viral properties. Modifications in the Hotoda sequence at the 5’-3’ ends led to the identification of molecules which bind to CCR5- binding sites on gp120. This binding inhibits the interaction between the viral gp120 and the CD4+ receptors, thus preventing viral entry, making these molecules potential targets for use in HIV/AIDS therapy [95]. 

#### 5.2.2. Aptamers Targeting Reverse Transcriptase, RNase H and Integrase

Reverse transcriptase (RT) is responsible for two functions: firstly, DNase polymerase activity, converting RNA to DNA or vice-versa. After membrane fusion and de-capsulation, RT converts viral RNA to a complementary DNA (cDNA), which can then be integrated in the cells’ genome. Inhibition of RT halts viral infectivity and its progression. Therapeutic FDA approved drugs such as zidovudine, nevirapine, and stavudine [138] with this function are available but these are less effective on their own and therefore require administration in conjunction with one another. They are very effective in preventing viral replication for some time but since reverse transcriptase lacks proof reading abilities, mutations develop resulting in drug resistance. Secondly, RNase H activity in which RNase H functions in dsRNA cleavage before the action of RT. Following the RNA cleavage and reverse transcription, the viral genome is then integrated into the host cell’s genome through a process catalysed by integrase (IN) [138,139,140].

Aptamers are superior potential solutions to drug resistance and a number of studies have demonstrated their potential therapeutic features. In 2016, Gonzalez and colleagues reported the discovery of a 37NT DNA aptamer that was found as highly specific and able bind to HIV-1 RT. When tested on the HIV-1 HXB strain, the aptamer blocked the enzyme’s active site, thereby inhibiting template binding and consequently preventing viral replication. Other sets of aptamers called ODNs 93 and 112, known to have G-rich sequences, were found to possess inhibitory properties against HIV-1 RNase H activity of HIV-1 RT in the nanomolar range in vitro. Interestingly, derivatives of ODNs 93 and 112, named ODNs 93 del and 112 del, with shorter sequences, were still found to be effective and stable since they sustained their ability to form G4-structures. The derivatives were found to inhibit HIV-1 integrase, also in the same nanomolar range [95,141].

#### 5.2.3. Aptamers Targeting Nucleocapsid Protein (NC)

One survival mechanism exhibited by the HIV virus that has led to its extensive success is its mutating ability. This is possible through certain conserved proteins, one of which is known as the nucleocapsid protein (NC). This protein is majorly responsible for viral replication due to its chaperoning activity by means of its interaction with nucleic acids resulting in the unfolding of the secondary structure of viral RNA [142]. Inhibiting the NC protein would in turn impede viral progression and result in a non-infectious virus hence; the protein is considered a target for anti-viral therapy. It has been suggested that stabilized RNA aptamers could function as inhibitors for the viral packaging of HIV. This is based on a study where RNA aptamers with high affinity were selected and isolated from an RNA library which exhibited competitive binding with the psi RNA binding to the NC protein [143].

### 5.3. Aptamers in Hepatitis Virus

Available treatments for HBV include adenofovir, lamivudine and entecavir. Apart from the side effects demonstrated by these drugs, mutations that occur as a result of the infidel nature of reverse transcriptase are the cause of drug resistance [144]. Drug limitations increases the significance of the search for other modes of therapy that produce no side effects such as aptamers which are non-immunogenic and are therefore better agents for therapeutic applications. In 2008, Feng and colleagues [145] selected two aptamers (S1 and S2) against reverse transcriptase P protein; they subjected the aptamers to complete DHBV genomes PCD16, which are regulated by a CMV promoter. These were then transfected into LMH cells to determine potential roles in the virus’s replication cycle. S2 could strongly bind and inhibit P protein and hence prevent DNA synthesis and capsid formation. The binding of S1 on the other hand, displayed no effect on the formation of the capsid [145]. Following this study, Feng et al. [115] selected two aptamers; S6 and S9, and found that these aptamers are specific and have high affinity for Mini P (protein) in vitro. They further introduced S9 to HBV infected HepG2 cells and the observations revealed a great suppression of pgRNA and DNA synthesis. Additionally, S9 could successfully bind and inhibit P protein, thus preventing its association with pgRNA and therefore inhibiting viral replication.

Hepatitis C related drug resistance is an accumulating problem [146] and fortunately, studies have increasingly demonstrated that aptamers show great potential for use in the road to discovering specific therapeutic agents for this infirmity. Lee and co-workers [116] synthesized two aptamers; 2′ hydroxyl RNA aptamer and 2′ fluoro RNA aptamer against NS5B of HCV and tested the aptamers on whole cells. The 2-OH RNA and 2-F RNA aptamers were found to bind specifically and inhibit the replicase activity of NS5B, hence preventing viral replication. Later, Wandtke et al. [94] reported the selection of an aptamer that is highly specific to the E2 glycoprotein of HCV, a co-receptor of human CD81 observed on hepatocytes and B-lymphocytes. The ZE2 aptamer functions as a complete inhibitor of E2 and when tested on human established cell lines of hepatocellular carcinoma, it was noted for decreasing viral RNA and reducing E2 protein levels following verification by reverse transcription—qPCR (qpT-PCR) and western blot [94].

### 5.4. Aptamers in Rubella and Schistosomiasis

Despite the present vaccines and treatment of measles, emerging drug resistance remains the problem and the use of Veraptus therapeutic aptamers against infectious viral diseases, have been reported and serve as potential solutions to drug resistance [147]. Nevertheless, more aptamer work still needs to be done as an alternative method for therapy.

Among other schistosomiasis therapeutic agents, praziquantel has been the most reliable drug used for the control of the disease and is effective on all three strains of the main *Schistosoma* spp. [148,149]. However, praziquantel is slowly losing its value due to drug resistance and its noted ineffectiveness against the juvenile form of the worm [150]. The exact mechanisms by which these helminths resist PZQ are still not understood and this drug resistance seriously declares the need for novel therapeutic agents such as aptamers which will be specific and highly effective in retarding disease progression and hence controlling its spread. The LC15 aptamer that was discovered by Long et al. [123] demonstrated specificity for *S. japonicum* eggs and highlighted aptamers as potential agents that could act as drug-carriers that ensure specificity and hence provide improved and alternative forms of therapeutic agents [151]. 

## 6. Conclusions and Future Perspectives

The acceptance of certain aptamers such as Macugen^®^ for use in the pharmaceutical industry with others going through phase trials mark the aptitude of aptamers. Aptamers are highly non-immunogenic, are specific to their targets and very sensitive, thus they succeed in being able to distinguish between isoforms of the same target. Hence, to address the problems encountered by diagnostic tools, they can therefore be used as a basis for diagnostic tools that will ensure early detection of diseases without any waiting period before definite diagnosis. This will greatly improve the time of diagnosis and enable good and better disease control. Aptamer structures can be modified easily for functional enhancement and stability, and these functions suggest that they can be used in conjugation with any therapeutic agent, directing the drug to specific sites and improving drug specificity while combating drug resistance in the process. Many studies have demonstrated the potential of aptamers as shown in Table 2, but there is still a gap in the search for aptamers against Rubella virus. Taken together, aptamers are the future and their application will be enhanced even for evolving diseases since they can be synthesized within short periods and in high batches.

## Figures and Tables

**Table 1:** Strengths and limitations of aptamer technology.

Strengths	Limitations
Aptamers’ entire selection process can be carried out in a test tube	Unmodified RNA aptamers are susceptible to nuclease degradation in in vivo applications [24]
They have high binding affinities, selectivity and specificity to their targets making them important for diagnostics and targeted therapy	Unmodified oligonucleotides have a short half-life, i.e., they quickly undergo renal filtration [24]
They are thermostable [39]	
Can be structurally modified to enhance specificity and stability, therefore increasing aptamer half-life in in vivo applications [39,40,41]	
They are non-toxic and elicit little or no immunogenic response [41]	

**Table 2:** Molecular Application of Aptamers in Diseases.

Aptamers	Sequence 5’	Structure	Organism	Target	Function
AS1411 (DNA)	d(GGTGGTGGTGGTTGTGGTGGTGGTGG)	G-quadruplex	Cancer	Nucleolin	Specifically detects cancer cells [14,48] Promotes cell death, prevents cell proliferation via Rac 1 activation [66] Up-regulates Galectin-14 and suppresses hepatocellular carcinoma [68]
T40214 (DNA)	d(GGGCGGGCGGGCGGGC)	G-quartet	Cancer	STAT3- protein	[14,79,152]
HJ24 (DNA)	d(AGCGTCGAATACCACACGGGGGTTTTGGTGGGGGGGGCTGGGTTGTCTTGGGGGTGGGCTAATGGAGCTCGTGGTCAT)	G-quadruplex	Cancer	Shp2	In vitro studies (IC50 = 29 nM) [14]
TTA1 (RNA)	-	-	Cancer	Domain of tenacin	Prevents angiogenesis, invasion and tumour growth [48]
MUC 1 (DNA)	GCAGTTGATCCTTTGGATACCCTGG	-	Cancer	Mucin-1	[26]
AS1411 (DNA)	d(GGTGGTGGTGGTTGTGGTGGTGGTGG)	G-quadruplex	HIV	Nucleolin	Antiviral activity (in vitro) (EC_50_ = 15.4 nM) [14]
37NT (DNA)	-	-	HIV (HXB strain)	HIV reverse transcriptase (RT)	Blocks RT active site, prevents viral replication [95]
ISIS5320	d(T*T*G*G*G*G*T*T)	G-quadruplex	HIV	HIV gp120	Prevents viral adsorption, inhibits HIV infection (in vitro) IC50 = 0.30 μM) [14]
Hotoda sequence	DBB-d(TGGGAG) and TBDPS-d(TGGGAG)	G-quadruplex	HIV	HIV gp120	In vitro (IC_50_ = 0.37 μM and 0.88 μM, resp.) [14]
Zintevir	d(G*TGGTGGGTGGGTGGG*T)	G-quadruplex	HIV	HIV Integrase	Completed phase 1 [137]
ODN93	d(GGGGGTGGGAGGAGGGTAGGCCTTAGGTTTCTGA)		HIV	HIV RNase H	Inhibits RNase H activity In vitro (IC_50_ = 0.50 μM) [14,95]
ODN 112	d(CCAGTGGCGGGTGGGTGGGTGGTGGGGGGACTTGG)		HIV	HIV RNase H	Inhibits RNase H activity In vitro (IC_50_ = 0.50 μM) [14,95]
ZE2 (DNA)	-	-	HCV	HCV-E2 glycoprotein	Inhibits E2 binding on CD81 receptors [12]
Apt.No.28 (DNA)	-	-	HBV	HBV nucleocapsid	Prevents nucleocapsid assembly and DNA synthesis [153]
NK2 (DNA)	-	-	Mycobacterium tuberculosis H37Rv strain	Membrane Proteins	Acts as an antibacterial agent, promotes cytokine production [12]
LC6 and LC15 (DNA)	-	-	Schistosomiasis	Schistosoma eggs	Binds specifically to Schistosoma eggs [121]

NALATS: Not Available in Literature as at the time of search [-].
